# Supplementary material for: Serum metabolite profiles are associated with the presence of advanced liver fibrosis in Chinese patients with chronic hepatitis B viral infection
Source: BMC Med. 2020 Jun 5;18:144. doi: 10.1186/s12916-020-01595-w (PMC7273661; doi:10.1186/s12916-020-01595-w)
Supplement: Supplementary file 1 — Additional file 1. Fibrosis and Cirrhosis Patients with hepatitis B viral (HBV) Infection and Normal Controls (Cohort 1). Patients with CHB-induced Fibrosis and Cirrhosis and normal controls (Validation Set, Cohort 2). Inclusion and exclusion criteria for patients with chronic HBV infection. Exclusion criteria for normal controls. Medication the patients received at the time of sampling. Quality of care. Measurement of bile acids. Measurement of Amino Acids. Measurement of FFAs. Quality Control Procedure. [file 12916_2020_1595_MOESM1_ESM.docx]

**Additional file 1:**

**Fibrosis and Cirrhosis Patients with hepatitis B viral (HBV) Infection and Normal Controls (Cohort 1)**

504 patients diagnosed with chronic hepatitis B virus (CHB)-induced fibrosis and cirrhosis, ages 15-75, were recruited at Shuguang Hospital affiliated to Shanghai University of Traditional Chinese Medicine (Shanghai, China) from April 2013 to June 2015. All the patients were tested positive for HBV-DNA or positive for hepatitis B surface antigen (HBsAg). Infection with chronic HBV was diagnosed according to the “Guideline on prevention and treatment of chronic hepatitis B in China”. All patients were clinically stable at the time of assessment and received a liver biopsy directed by ultrasonography within 1 week after inclusion in the study except those diagnosed as having decompensated cirrhosis, details on inclusion and exclusion criteria are provided in a later section. The patients diagnosed as early stage fibrosis (S0-S2) were newly diagnosed and the blood samples were collected before receiving any drugs or antivirals. About 44% patients with S3 and S4 were administered entecavir.

502 participants were recruited as normal controls from the Physical Examination Center of Shuguang Hospital*.* There were no significant differences in age, sex, and BMI between normal controls and patient group (Table S1). Routine biochemical tests were conducted to ensure that the study subjects were free of inflammation and metabolic diseases. B-mode ultrasound examination was performed to exclude participants with fatty liver.

The study was approved by the institutional review board of the Shuguang Hospital affiliated to Shanghai University of Traditional Chinese Medicine. All participants signed informed consent forms for the study.

Overnight (12 hr) fasting blood specimens were collected for hematological and routine biochemical tests and metabolomic analyses of bile acids (BAs), free fatty acids (FFAs) and amino acids (AAs).

**Patients with CHB-induced Fibrosis and Cirrhosis and normal controls (Validation Set, Cohort 2)**

The results obtained from the training set were validated in an independent cohort of patients (Cohort 2), including 141 CHB-induced fibrosis and 159 CHB-induced cirrhosis and 90 normal controls, independently recruited from Xiamen Hospital of Traditional Chinese Medicine. All patients were received a liver biopsy directed by ultrasonography within 1 week after inclusion in the study as performed in Cohort 1subjects. We quantitated serum BAs, AAs and FFAs in this validation set using the same procedure as before.

Inclusion and exclusion criteria were the same as those for the samples in Cohort 1 (see below). All participants provided informed consent prior to the study and the protocol was approved by the institutional review board of the hospital.

**Inclusion and exclusion criteria for patients with chronic HBV infection**

**Inclusion criteria**:

(1) Age 15-75 years old

(2) Clinically diagnosed with chronic hepatitis B (CHB) and post-hepatitis B liver fibrosis and cirrhosis according to the “Guideline on prevention and treatment of chronic hepatitis B in China (2010)”.

(3) Able to read and understand the language used in the Informed Consent, capable of understanding the study requirements and willing to comply with the research procedures.

(4) Able to provide written/verbal consent/assent.

**Exclusion criteria**:

(1) Younger than 15 years or older than 75 years

(2) Co-infection with human immunodeficiency virus (HIV), hepatitis C virus (HCV), hepatitis D virus (HDV), or hepatitis E virus (HEV), or anti-HAVIgM, or anti-HCV, or anti-HDV, or anti-HEV, or anti-HIV positive

(3) Other causes (include fatty liver or drug) induced fibrosis and cirrhosis

(4) Other chronic disease requiring daily medication (e.g. diabetes)

(5) In combination with severe cardiovascular, pulmonary, renal, endocrine and primary hematopoietic system disease

(6) Pregnant or breast-feeding women

(7) Chronic biliary liver diseases such as primary biliary cholangitis, primary sclerosing cholangitis or primary biliary cirrhosis

(8) Hepatic schistosomiasis patients

(9) Liver transplant, gastrointestinal disorders

(10) Recently participated (within 6 months) or who were currently participating in other clinical trials

(11) Psychoactive medication use

(12) Evidence of drug addiction within the past one year prior to enrollment

(13) Alcohol consumption within 6 months and either antibiotic or probiotic/prebiotic use within the last 6 weeks

(14) Unable to finish/fill in the questionnaire

(15) Patients who were unable to provide fasting blood samples and those unable to complete the study

(16) Diagnosed with hepatic carcinoma (HCC) according to “clinical diagnosis and staging criteria of primary hepatic carcinoma”.

**Exclusion criteria for normal controls**

Individuals were excluded from participation if they were older than 75 years or younger than 15 years of age, were pregnant or lactating, had significant cardiopulmonary, renal, gastrointestinal disease or liver disease, acute or chronic infections, active malignancy, other acute or chronic diseases requiring treatment, prescription medication use within the previous 2 months.

**Medication the patients received at the time of sampling**

The patients with S3 and S4 received an oral dose of 0.5 mg/day of entecavir before meal or at least 2 hours after meal.

**Quality of care**

The participants received high-quality care in the individual hospitals involved in this study. First, the hospitals involved in this study are categorized as “first-line 3A hospitals”, which are the top-tier, tertiary hospitals (typically with a bed capacity exceeding 500) providing high quality care to patients with comprehensive medical programs. Second, the chronic liver disease patients in our study were diagnosed strictly following the “Guideline on prevention and treatment of chronic hepatitis B in China (2010)” by Chinese Society of Hepatology and Chinese Society of Infectious Diseases, Chinese Medical Association, and the “Guideline for the diagnosis and treatment of liver fibrosis with integrative medicine” by Liver Disease Committee, Chinese Association of Integrative Medicine. Thirdly, treatment options for each patient include standard anti-viral therapies as well as traditional Chinese medicine (TCM). The recommendations of the TCM application were not only based on the individual symptoms (TCM symptomatic pattern classification) and quality of evidence, but also on the advantages and disadvantages of the interventions, the variability of patient conditions and preferences, and the reasonable utilization of medical and financial resources. At present, there is no effective anti-fibrotic therapeutic agent available clinically. TCM, however, has a unique role in the treatment of hepatic fibrosis. A number of TCM formulas have shown to have anti-hepatic fibrosis effects and are currently being used in clinical treatment of liver fibrosis in China. The most widely used herbal formula preparations include Fuzhenghuayu capsules, Anluohuaxian pills and Fufang Biejia Ruangan tablets. All these preparations were made available to individual patients in hospitals involved in our study based on the TCM recommendations, in addition to standard antiviral treatments.

**Measurement of bile acids**

***Serum sample preparation.*** An aliquot of 50 μl of serum was mixed with 150 µl of methanol (containing 0.10 μM of lithocholate-2,2,4,4-D4 (LCA-D4), ursodeoxycholate-2,2,4,4-D4 (UDCA-D4), and cholate-2,2,4,4-D4 (CA-D4) from C/D/N Isotopes Inc. as internal standards). The mixture was vortexed for 2 min, allowed to stand for 10 min, and then centrifuged at 20,000 g at 4 °C for 10 min. An aliquot of 160 µL of supernatant was transferred to a clean tube and vacuum dried. The residue was redissolved with equal amount of acetonitrile (0.1% formic acid) and water (0.1% formic acid) to a final volume of 40 μL. After centrifugation, the supernatant was used for UPLC-MS/MS analysis.

***Method validation****.* Each aliquot of the 41 standard stock solution included: cholate(CA), glycocholate (GCA), taurocholate (TCA), chenodeoxycholate (CDCA), glycochenodeoxycholate (GCDCA), taurochenodeoxycholate (TCDCA), deoxycholate (DCA), glycodeoxycholate (GDCA), taurodeoxycholate (TDCA), ursodeoxycholate (UDCA), glycoursodeoxycholate (GUDCA), tauroursodeoxycholate (TUDCA), lithocholate (LCA), glycolithocholate (GLCA), taurolithocholate (TLCA), hyocholate (HCA), glycohyocholate (GHCA), taurohyocholate (THCA), α-muricholate (αMCA), tauro-α-muricholate (TαMCA), β-muricholate (βMCA), tauro-β-muricholate (TβMCA), ω-muricholate (ωMCA), tauro-ω-muricholate (TωMCA), hyodeoxycholate (HDCA), glycohyodeoxycholate (GHDCA), taurohyodeoxycholi acid (THDCA), murocholate (MuroCA), dehydrocholate (DHCA), glycodehydrocholate (GDHCA), taurodehydrocholate (TDHCA), 3-ketocholate (3-KCA), 7-ketodeoxycholate (7-KDCA), isodeoxycholate (isoDCA), apocholate (ACA), 6-ketolithocholate (6-KLCA), 7-ketolithocholate (7-KLCA), 12-ketolithocholate (12-KLCA), 12-Ketochenodeoxycholate (12-KCDCA), 23-nordeoxycholate (23-NDCA), and isolithocholate (iso-LCA) [obtained from Steraloids Inc. (Newport, RI)] was mixed to obtain a mixed stock solution. Calibration solutions containing all 41 bile acid standards were prepared at a series of concentrations of 0.610, 1.221, 2.441, 4.883, 9.766, 19.531, 39.063, 78.125, 156.250, 312.5, 625.0, 1250.000, and 2500.00 ng/mL in naïve pooled serum depleted of bile acids using activated charcoal. The calibration curve and the corresponding regression coefficients were obtained by internal standard adjustment (data were normalized by isotope labeled internal standards).

***Bile acid quantitation***. Serum bile acids were measured using a Waters ACQUITY ultra performance liquid chromatography system equipped with a binary solvent delivery manager and a sample manager (Waters, Milford, MA) coupled with a Waters XEVO TQ-S mass spectrometry with an ESI source (Waters, Milford, MA). The entire UPLC–TQMS system was controlled by MassLynx 4.1. All chromatographic separations were performed with an ACQUITY BEH C18 column (1.7 µm, 100 mm × 2.1 mm internal dimensions) (Waters, Milford, MA).

Optimal LC conditions were as follows: The samples were eluted with the mobile phase consisting of 0.1% formic acid in LC-MS grade water (mobile phase A) and 0.1% formic acid in LC-MS grade acetonitrile (mobile phase B) and ran at a flow rate of 0.3 mL/min. The flow rate was 0.45 mL/min with the following mobile phase gradient: 0-1 min (5% B), 1-5 min (5-25% B), 5-15.5 min (25-40% B), 15.5-17.5 min (40-95% B), 17.5-19 min (95% B), 19-19.5 min (95-5% B), 19.6-21 min (5% B). The column was maintained at 45 °C and the injection volume for all samples was 5 µl.

The mass spectrometer was operated in negative ion mode with a 1.2 kv capillary voltage, a source and desolvation gas temperature at 150 and 550 °C, respectively. The data was collected with multiple reaction monitor (MRM) and the cone and collision energy for each bile acid used the optimized settings obtained from QuanOptimize application manager (Waters Corp., Milford, MA).

**Measurement of Amino Acids**

The serum levels of amino acids were analyzed by ultra-performance liquid chromatography triple quadruple mass spectrometry (UPLC-TQ/MS, Waters, Milford, MA, USA). A 40 μL aliquot of serum sample was mixed with 10 μL of isotope labeled internal standard (1 mM of each corresponding isotope labeled amino acid standards). After dilution with 80 μL of water, the sample was extracted with 500 μL of a mixture of methanol and acetonitrile (1:9, v/v). The extraction procedure was performed at −20 °C for 10 min after 2 min vortexing and 1 min ultrasonication. The sample was then centrifuged at 4 °C at 12000 rpm for 15 min. An aliquot of the 20 μL supernatant was vacuum-dried at room temperature. After that, the residue was redissolved by 100 μL of a mixture of methanol and water (1:1, v/v) with 1 μg/mL of L-2-chlorophenylalanine followed by the same vortexing, ultrasonication and centrifugation steps ahead. A volume of 80 μL supernatant was trasferred into the sampling vial for UPLC-TQ/MS analysis (Waters, Manchester, U.K.). A 5 μL aliquot of sample was injected into an UPLC system (Waters, U.K.) with a 4.6 mm × 150 mm, 5 μm Eclipse XDB-C18 column (Agilent, USA). The column was held at 40 °C. The elution procedure for the column was 1% for the first 0.5 min,1–20% B over 0.5–9 min, 20–75% B over 9–11 min, 75–99% B over 11–16 min, and the composition was finally held at 99% B for 0.5 min, where A = water with 0.1% formic acid and B = acetonitrile with 0.1% formic acid and the flow rate was 0.4 mL/min. A Waters XEVO-Triple Quadrupole MS was used for the mass spectrometry detection. The temperature for the source and desolvation gas was set at 150 and 450 °C respectively. The gas flow for cone and desolvation was 50 and 800 L/h respectively. The capillary voltage was set to 3.0 kV. All the compounds were detected in MRM mode.

The calibration curve and the corresponding regression coefficients for each metabolite were obtained by internal standard adjustment (data were normalized by isotope labeled internal standards).

**Measurement of FFAs**

All the serum specimens were stored at −80 °C until analyzed. Each sample aliquot of 40 μL was mixed with 10 μL of isotope labeled internal standard (5 μg/mL C19:0-d37), and 500 μL of isopropyl/hexane (v/v=80/20) with 2% phosphate (2M). The mixture was extracted with 400 μL of hexane and 300 μL of water. After centrifugation, an aliquot of 400 μL of supernatant was transferred to an Eppendorf microcentrifuge tube and the remaining mixture was further extracted with an additional 400 μL of hexane. After centrifugation, an aliquot of 500 μL of supernatant was combined with the first supernatant and dried under vacuum. The residue was reconstituted with 80 μL of methanol, filtered with 0.22-μm membrane (EMD Millipore, Billerica, MA) and then analyzed using UPLC-QTOF-MS.

The set-up parameters for the UPLC-QTOF-MS analysis were as follows. A BEH C18 (2.1 mm × 100 mm, 1.7 μm) chromatographic column was used for separation with column temperature set at 40 °C. The elution solvents were water (A) and acetonitrile/isopropyl (v/v = 80/20, B) with a flow rate of 400 μL/min. The initial gradient was 70% B and kept for 2 min; increased to 75% B within 3 min; increased to 80% B in 5 min; increased to 90% in 3 min; increased to 99% within 3 min and kept at 99% for 5 min before switching back to initial condition. The MS was operated at a negative electrospray ionization mode with a capillary voltage of 2.5 kV. The sample cone and the extraction cone were set at 55 V and 4 V, respectively. The source temperature was set to 150 °C, and the desolvation temperature was set to 450 °C with a desolvation gas flow rate of 650 L nitrogen per hour.

The calibration curve and the corresponding regression coefficients for each metabolite were obtained by internal standard adjustment (data were normalized by isotope labeled internal standards).

**Quality Control Procedure**

In addition to the internal standards used for quality control (QC), another two types of QC samples, including test mixtures (a group of commercially available standards with a mass range across the system mass range), and pooled biological samples were used for our metabolomic procedures. The QC samples were prepared along with the study samples and run after each 10 serum samples. The QC samples were kept at 10 °C during the entire analysis.

The QC samples were evenly inserted in each set of the analysis running sequence to monitor the stability of the large-scale analysis. The noise baseline was established from reagent blank measurement and any metabolite with signal to noise ratio ≤ 3.0 was rejected from statistical analysis. Relative standard deviations (RSDs) of each metabolite in the QC samples measured were calculated. The RSD for the QCs was less than 15% for each batch of sample analysis.
