# Supplementary material for: Serum metabolite profiles are associated with the presence of advanced liver fibrosis in Chinese patients with chronic hepatitis B viral infection
Source: BMC Med. 2020 Jun 5;18:144. doi: 10.1186/s12916-020-01595-w (PMC7273661; doi:10.1186/s12916-020-01595-w)
Supplement: Supplementary file 2 — Additional file 2: Figure S1. Representative H&E staining images of chronic liver disease patients with necro-inflammation activity at G0 (A), G1 (B), G2 (C), G3 (D) to G4 (E) according to the Scheuer’s classification. Scale, 200 μm. Figure S2. Representative Masson’s trichrome staining, collagen stained blue. Collagen portionate area increased significantly along with the degree of liver fibrosis (from S0 to S4, Table 1). Scale, 200 μm. Figure S3. PCA scores plot for CLD patients and normal controls using the identified four metabolite markers in training and validation sets. Figure S4. Correlation coefficient matrix among the four selected serum metabolites, previously proposed liver fibrosis markers, and clinical markers of chronic liver disease (fibrosis stages, necro-inflammation, and medication). Figure S5. 10-fold cross-validation AUROC and AUPR of machine learning methods and clinical indices. Figure S6. PCA scores plot for CLD patients of S0–2, S3 and S4 using the identified four metabolite markers in training and validation sets. Figure S7. Example decision trees from random forest models. (a) An example decision tree of Model 1. (b) An example decision tree of Model 2. (c) An example decision tree of Model 3. Figure S8. Micro-ROC and micro-PR of metabolite marker panel and clinical indicators in multi-group classification of S0–2 vs. S3 vs. S4. (a) micro-ROC and (b) micro-PR for the classification of S0-2 vs. S3 vs. S4 in Cohort 1. (c) micro-ROC and (d) micro-PR for the classification of S0-2 vs. S3 vs. S4 in Cohort 2. [file 12916_2020_1595_MOESM2_ESM.docx]

**Additional file 2:**

**Figure S1.** Representative H&E staining images of chronic liver disease patients with necro-inflammation activity at G0 (A), G1 (B), G2 (C), G3 (D) to G4 (E) according to the Scheuer’s classification. Scale, 200 μm.

**
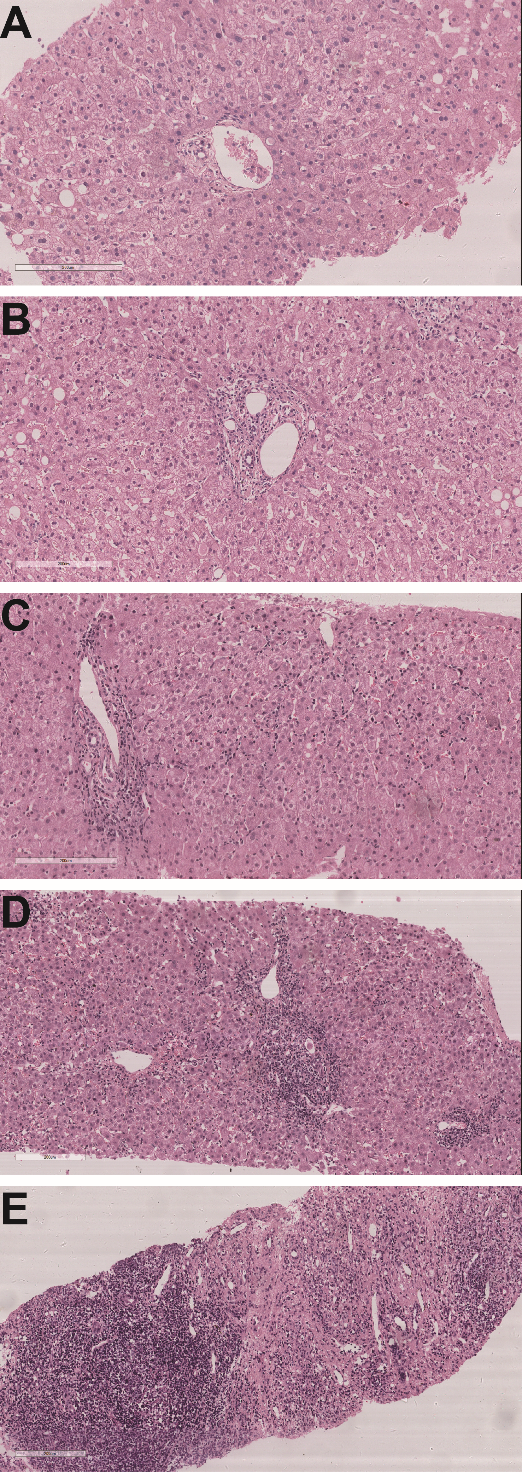
**

**Figure S2**. Representative Masson's trichrome staining, collagen stained blue. Collagen portionate area increased significantly along with the degree of liver fibrosis (from S0 to S4, Table 1). Scale, 200 μm.

**
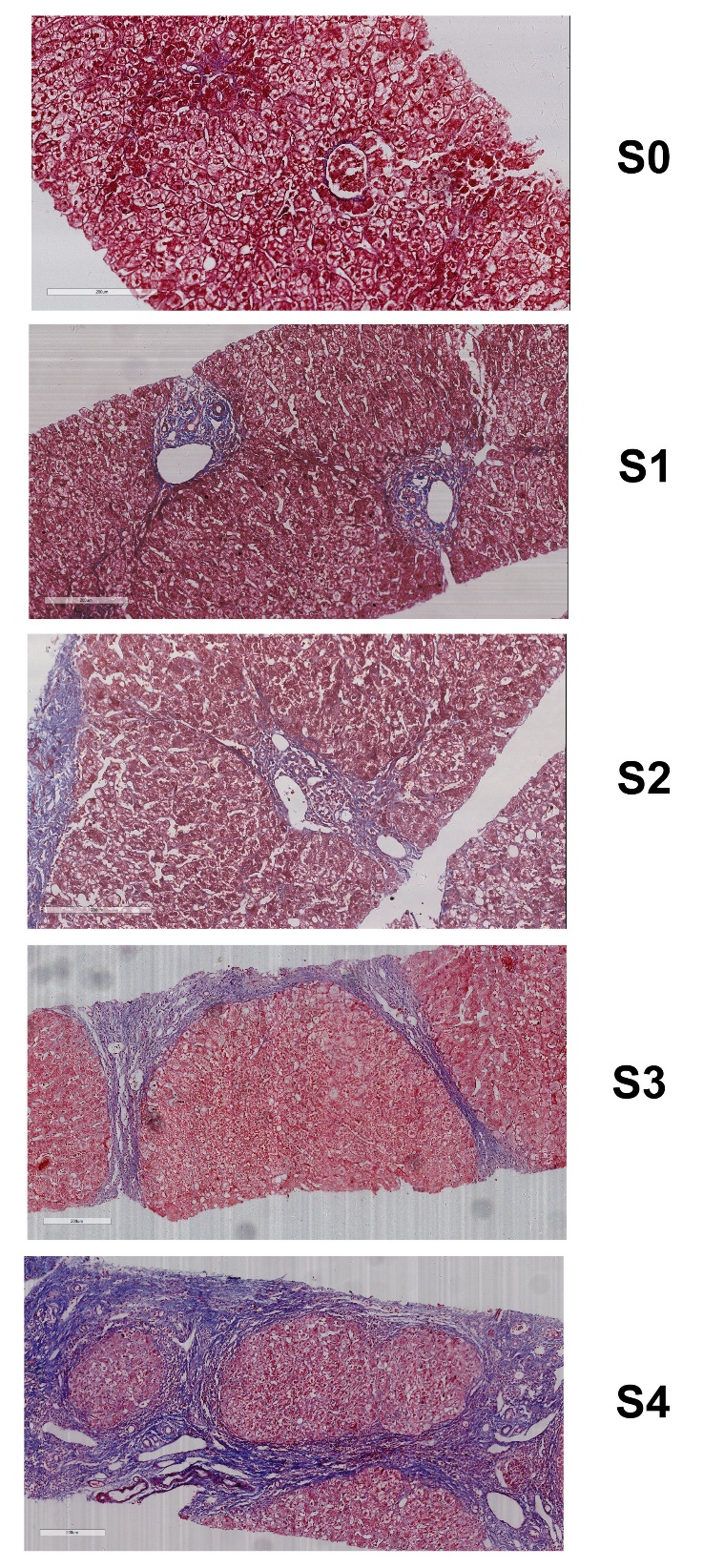
**

**Figure S3.** PCA scores plot for CLD patients and normal controls using the identified four metabolite markers in training and validation sets.

Note: “PC1” means the first principal component, “PC2” mean the second principal component. The percentage in the bracket means the percentage of the total variance explained by this principal component. The dash ellipses mean the 80% normal confidence ellipse.

**
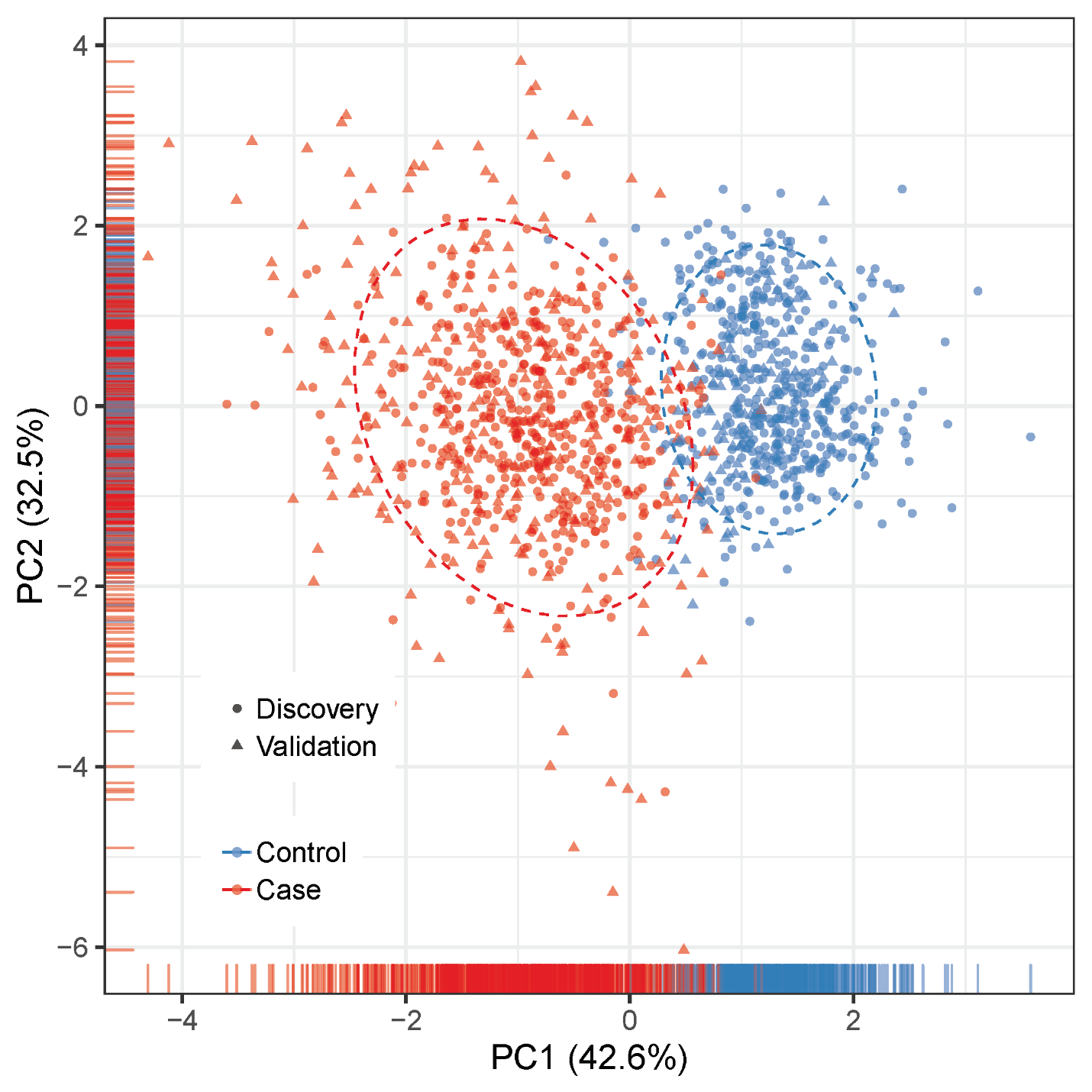
**

**Figure S4**. Correlation coefficient matrix among the four selected serum metabolites, previously proposed liver fibrosis markers, and clinical markers of chronic liver disease (fibrosis stages, necro-inflammation, and medication).

*, p < 0.05, **, p<0.01, ***, p<0.001, by Spearman correlation analysis.


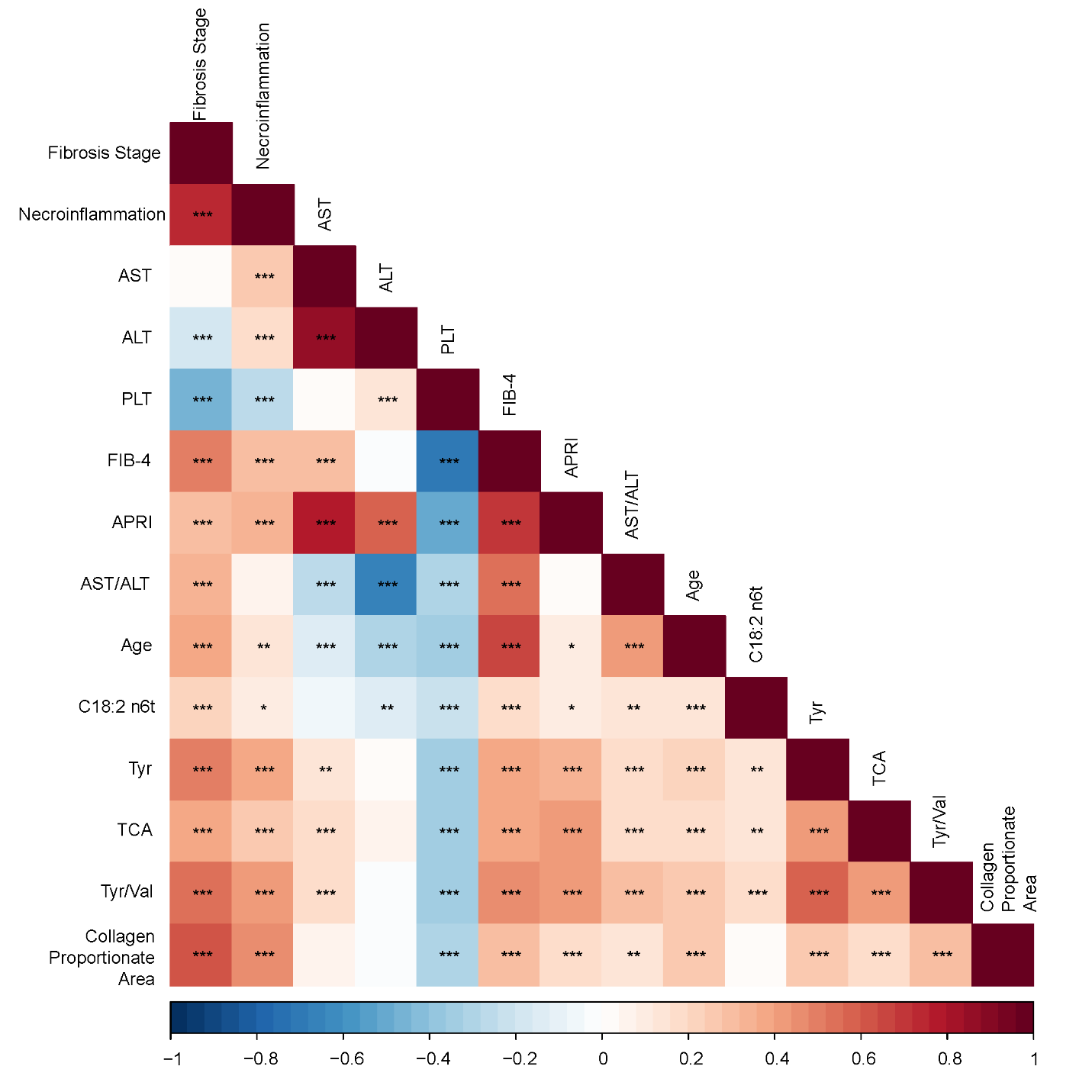


**Figure S5.** 10-fold Cross-Validation AUROC and AUPR of machine learning methods and clinical indices

(a) The CV AUROC and AUPR for the classification of CLD vs. Control. (b) The CV AUROC and AUPR for the classification of fibrosis vs. cirrhosis. (c) The CV AUROC and AUPR for the classification of early fibrosis vs. advanced fibrosis.


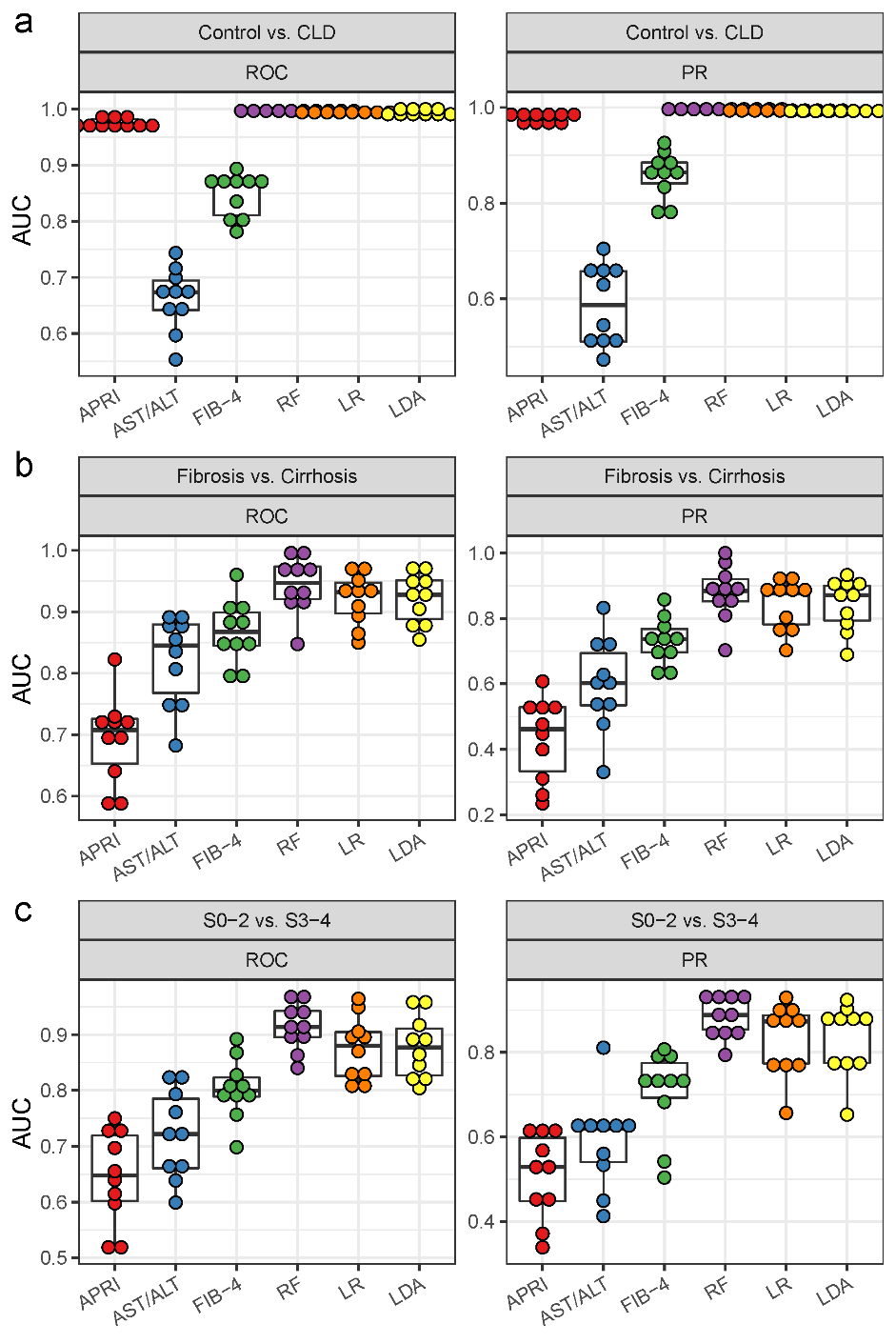


**Figure S6.** PCA scores plot for CLD patients of S0-2, S3 and S4 using the identified four metabolite markers in training and validation sets.

Note: “PC1” means the first principal component, “PC2” mean the second principal component. The percentage in the bracket means the percentage of the total variance explained by this principal component. The dash ellipses mean the 80% normal confidence ellipse.


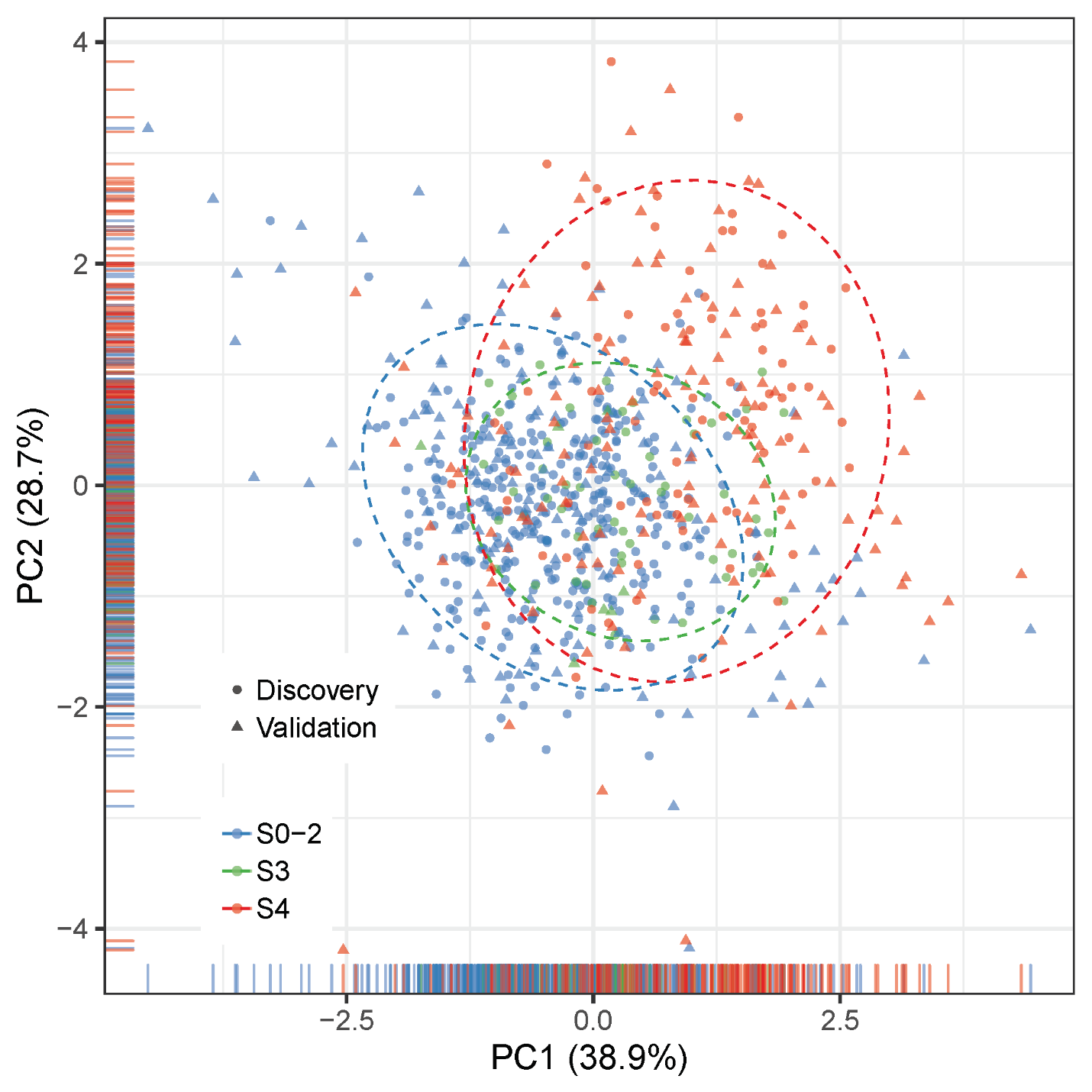


**Figure S7. Example decision trees from random forest models.** (a) An example decision tree of Model 1. (b) An example decision tree of Model 2. (c) An example decision tree of Model 3.

Note: Each node (except for leaf node) is a decision rule (e.g., Tyr/Val < 0.43 in panel c). Data was splited by each decision rule with yes to the left branch and no the right branch. Leaf nodes reprensent the final predicted outcomes. At each node, we can see a predicted class, a predicted probability of postive class, and a percentage of observations on this node. The color gradients from blue to green represent the predicted probabilities of positive class.


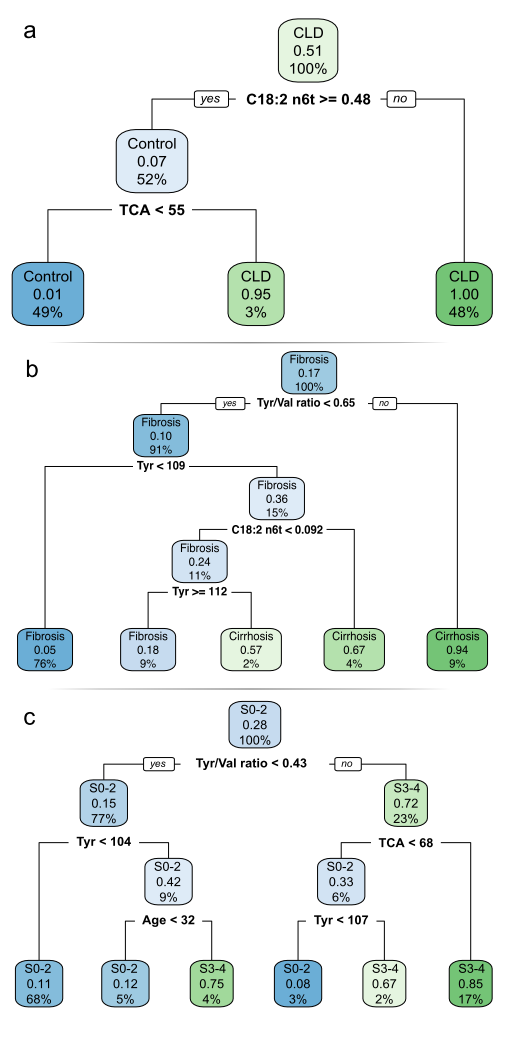


**Figure S8. Micro-ROC and micro-PR of metabolite marker panel and clinical indicators in multi-group classification of S0-2 vs. S3 vs. S4.** (a) micro-ROC and (b) micro-PR for the classification of S0-S2 vs. S3 vs. S4 in Cohort 1. (c) micro-ROC and (d) micro-PR for the classification of S0-S2 vs. S3 vs. S4 in Cohort 2.

**
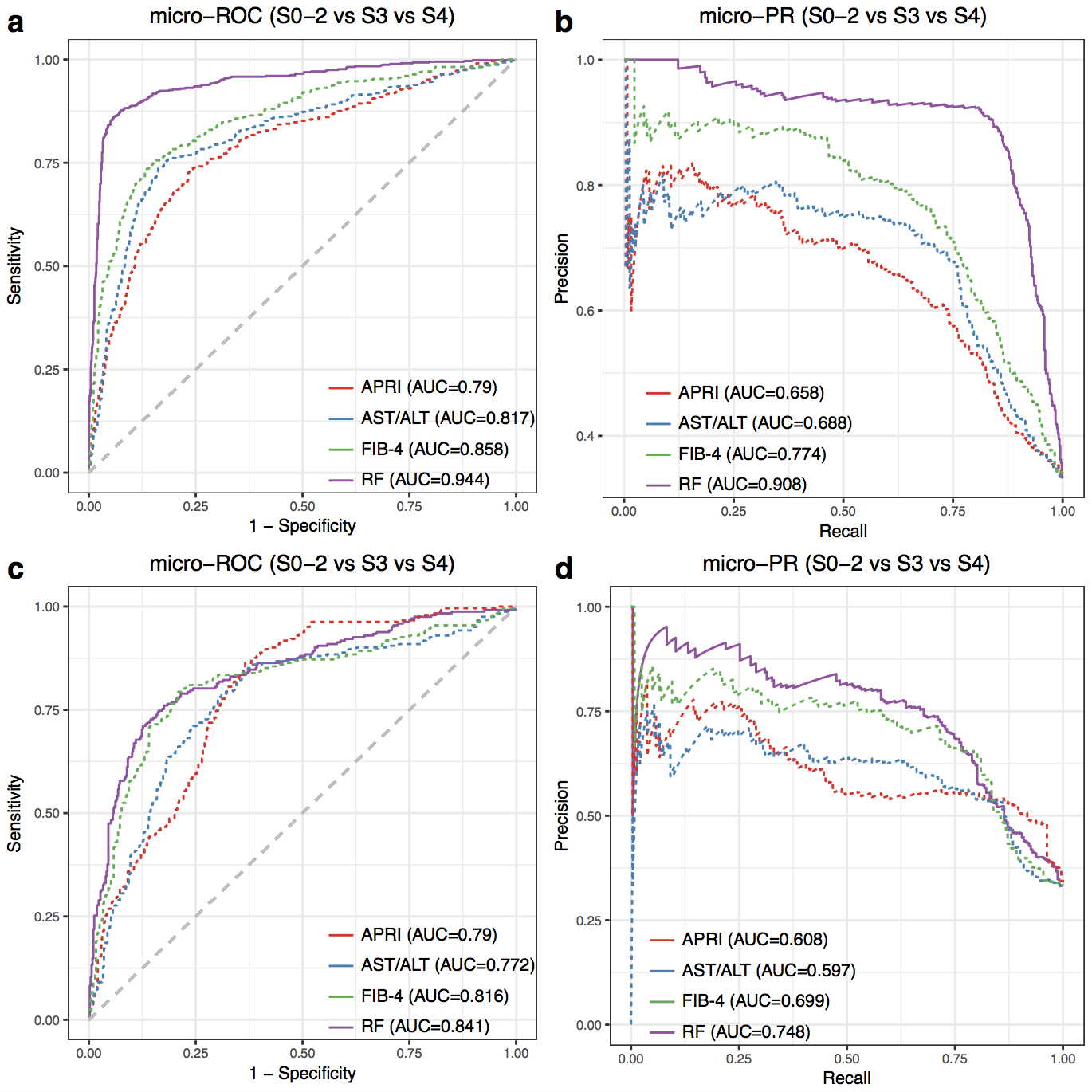
**
