# Supplementary material for: Serum metabolite profiles are associated with the presence of advanced liver fibrosis in Chinese patients with chronic hepatitis B viral infection
Source: BMC Med. 2020 Jun 5;18:144. doi: 10.1186/s12916-020-01595-w (PMC7273661; doi:10.1186/s12916-020-01595-w)
Supplement: Supplementary file 3 — Additional file 3 Table S1. Clinical data of patients with chronic liver disease (CLD) and normal controls in Cohorts 1 and 2. Table S2. Serum bile acid, free fatty acid, and amino acid concentrations in patients with chronic liver disease (CLD) and in normal controls in Cohorts 1 and 2. Table S3. Results for measurement of the metabolite marker panel, APRI, FIB-4, and ALT/AST ratio in the prediction of liver fibrosis using the optimal cut-off values generated using the cohort specific data from this study. Table S4. Logistic regression analysis of metabolite marker panel-based RF-score to discriminate patients with fibrosis from patients with cirrhosis and S0–2 with S3–4 adjusting with potential confounding variables. Table S5. Net reclassification improvement and integral discriminant improvement analyses comparing RF and other clinical indexes on validation sets. [file 12916_2020_1595_MOESM3_ESM.docx]

**Additional file 3:**

**Table S1**. Clinical data of patients with chronic liver disease (CLD) and normal controls in Cohorts 1 and 2.

| **Dataset** | **Cohort 1 training set** | | | | | | **Cohort 2 validation set** | | | | | |
| --- | --- | --- | --- | --- | --- | --- | --- | --- | --- | --- | --- | --- |
| **Group** | **Control** | **CLD** | **S0-2** | **S3-4** | **Fibrosis** | **Cirrhosis** | **Control** | **CLD** | **S0-2** | **S3-4** | **Fibrosis** | **Cirrhosis** |
| **Number** | 502 | 504 | 349 | 155 | 400 | 104 | 90 | 300 | 134 | 166 | 141 | 159 |
| **DBIL (μmol/L)** | 2.35±0.79 | 7.41±15.88*** | 4.94±6.95 | 13.66±26.77*** | 5.74±11.78 | 15.6±27.1** | 2.88±0.75 | 15.27±30.59*** | 8.86±19.05 | 16.98±32.86** | 7.8±17.12 | 17.93±33.82*** |
| **IBIL (μmol/L)** | 10.47±4.52 | 20.15±18.52*** | 17.05±10.28 | 27.99±29.38*** | 17.54±11.74 | 32.94±34.11*** | 11.09±2.99 | 20.12±19.54*** | 13.51±12.67 | 25.65±22.39*** | 13.62±12.37 | 26.11±22.8*** |
| **PALB (mg/L)** | 332.68±37.14 | 200.66±72.28*** | 214.1±69.25 | 165.58±68.37*** | 207.59±70.84 | 164.92±69.42*** |  | 145.76±88.2 | 171.56±89.02 | 99.41±65.19*** | 171.56±89.02 | 99.41±65.19*** |
| **CHE (KU/L)** |  | 7.5±2.19 | 8.17±1.63 | 5.69±2.47*** | 8.03±1.66 | 4.61±2.47*** |  | 6.28±6.71 | 6.63±2.34 | 5.79±10.05*** | 6.64±2.33 | 5.73±10.27*** |
| **Renal function** | | | | | | | | | | | | |
| **CREA (μmol/L)** |  | 72.53±18.02 | 72.5±14.62 | 72.59±24.87 | 72.52±14.33 | 72.55±30.68 | 73.4±14.43 | 68.98±44.76*** | 61.57±29.05 | 75.06±53.69** | 61.77±28.65 | 75.42±54.62* |
| **BUN (mmol/L)** | 4.82±1.03 | 3.88±1.62*** | 3.6±1.06 | 4.61±2.43*** | 3.63±1.07 | 5.16±2.89*** | 5.64±7.87 | 5.04±3.17 | 4.29±1.41 | 5.65±3.99*** | 4.3±1.4 | 5.7±4.05*** |
| **Blood lipids** | | | | | | | | | | | | |
| **CHOL (mmol/L)** | 5.18±0.94 | 4.64±1.18*** | 4.69±0.91 | 4.48±1.68 | 4.72±0.92 | 4.18±1.99* | 4.61±0.62 | 3.8±0.99*** | 4.11±0.96 | 3.5±0.93*** | 4.11±0.96 | 3.5±0.93*** |
| **TG (mmol/L)** | 1.56±0.82 | 1.23±1*** | 1.23±1.09 | 1.22±0.71 | 1.24±1.04 | 1.16±0.77 | 1.3±0.52 | 1.18±0.71** | 1.34±0.84 | 1.02±0.51*** | 1.32±0.83 | 1.02±0.51*** |
| **HDLC (mmol/L)** |  | 1.18±0.37 | 1.2±0.35 | 1.13±0.4 | 1.22±0.37 | 0.97±0.31*** | 1.36±0.48 | 1.17±0.43** | 1.23±0.44 | 1.11±0.42* | 1.23±0.43 | 1.11±0.43* |
| **LDLC (mmol/L)** |  | 2.97±0.89 | 2.98±0.86 | 2.95±0.97 | 3.02±0.86 | 2.73±1.03 | 2.64±0.53 | 2.2±0.86*** | 2.3±0.86 | 2.1±0.84* | 2.31±0.86 | 2.08±0.84** |
| **ApoAI (g/L)** |  | 1.41±0.35 | 1.46±0.29 | 1.27±0.43*** | 1.47±0.31 | 1.08±0.35*** |  | 1.28±0.39 | 1.36±0.38 | 1.12±0.38*** | 1.36±0.38 | 1.12±0.38*** |
| **ApoB (g/L)** |  | 0.96±0.25 | 0.95±0.25 | 0.96±0.28 | 0.97±0.25 | 0.89±0.27 |  | 0.71±0.27 | 0.78±0.24 | 0.6±0.27*** | 0.78±0.24 | 0.6±0.27*** |
| **Blood glucose** |  |  |  |  |  |  |  |  |  |  |  |  |
| **GLU (mmol/L)** | 5.35±0.7 | 5.25±1.24 | 5.14±0.81 | 5.57±1.99* | 5.17±0.9 | 5.74±2.39 | 4.9±0.64 | 6.84±21.87*** | 8.11±31.81 | 5.71±2.24 | 8.01±31.09 | 5.7±2.24 |
| **Coagulation function** | | | | | | | | | | | | |
| **PT (Sec)** |  | 13.64±2.67 | 12.89±0.97 | 15.55±4.21*** | 12.97±1.04 | 16.9±4.86*** |  | 14.25±2.7 | 12.92±1.21 | 15.38±3.08*** | 12.92±1.22 | 15.48±3.09*** |
| **PTR (%)** |  | 0.99±0.1 | 0.98±0.08 | 1.05±0.13*** | 0.98±0.08 | 1.09±0.16*** |  | 1.17±0.14 | 1.11±0.11 | 1.26±0.14*** | 1.11±0.11 | 1.26±0.14*** |
| **APTT (Sec)** |  | 37.72±6.48 | 36.27±3.79 | 41.38±9.69*** | 36.4±3.93 | 44.1±11.06*** |  | 35.36±12.68 | 32.62±8.62 | 39.36±16.18*** | 33.8±12.55 | 37.9±12.56*** |
| **TT (Sec)** |  | 17.67±4.89 | 16.28±1.52 | 21.17±7.86*** | 16.4±1.49 | 23.8±9.17*** |  | 18.62±1.89 | 18.47±1.59 | 18.85±2.25 | 18.41±1.6 | 18.96±2.26 |
| **FIB (g/L)** |  | 2.74±0.65 | 2.78±0.66 | 2.58±0.61** | 2.74±0.65 | 2.65±0.73 |  | 2.23±0.67 | 2.26±0.56 | 2.2±0.81 | 2.27±0.56 | 2.18±0.82 |
| **INR (%)** |  | 1.02±0.19 | 0.97±0.1 | 1.15±0.28*** | 0.98±0.11 | 1.23±0.32*** |  | 1.19±0.24 | 1.08±0.11 | 1.29±0.28*** | 1.08±0.11 | 1.3±0.28*** |
| **Blood tests** | | | | | | | | | | | | |
| **RBC (10^12/L)** | 4.75±0.43 | 4.55±0.67*** | 4.72±0.49 | 4.12±0.84*** | 4.71±0.51 | 3.79±0.82*** |  | 4.17±0.77 | 4.49±0.6 | 3.9±0.79*** | 4.5±0.62 | 3.86±0.77*** |
| **WBC (10^9/L)** | 6.8±1.67 | 5.4±1.68*** | 5.53±1.4 | 5.05±2.21* | 5.56±1.51 | 4.59±2.2*** |  | 4.88±2.09 | 5.18±1.84 | 4.64±2.24*** | 5.13±1.83 | 4.67±2.27** |
| **HCT (%)** | 44.32±3.58 | 42.2±5.29*** | 43.3±4.33 | 39.42±6.38*** | 43.26±4.29 | 37.04±6.57*** |  | 39.53±6.81 | 41.53±6.45 | 36.72±6.32*** | 41.5±6.35 | 36.39±6.36*** |
| **HGB (g/L)** | 141.21±18.45 | 138.48±22.94* | 144.5±15.29 | 123.1±30.79*** | 144.25±15.03 | 110.26±32.31*** |  | 131.14±22.19 | 138.7±18.89 | 117.55±21.31*** | 138.7±18.89 | 117.55±21.31*** |
| **MCH (pg)** | 28.04±1.58 | 30.83±3.63*** | 30.93±3.81 | 30.57±3.13 | 30.97±3.7 | 30.17±3.24 |  | 31.29±3.07 | 31.26±2.65 | 31.34±3.61 | 31.18±2.69 | 31.47±3.61 |
| **MCHC (g/L)** | 353.2±9.1 | 333.7±16.52*** | 332.75±16.42 | 336.13±16.6* | 332.68±15.58 | 338.7±19.88* |  | 337.68±19.03 | 337.11±23.28 | 338.48±10.53 | 337.3±22.75 | 338.28±10.76 |
| **MPV (fL)** | 8.26±0.72 | 9.79±8.61*** | 9.53±7.81 | 10.46±10.37 | 9.94±9.43 | 9.07±1.25 |  | 10.52±13.18 | 11.02±17.24 | 9.82±1.58 | 10.97±16.81 | 9.8±1.56 |
| **PDW (%)** | 15.27±0.77 | 16.57±0.74*** | 16.6±0.59 | 16.51±1.03 | 16.6±0.6 | 16.43±1.22 |  | 20.41±21.23 | 16.73±0.62 | 23.18±27.93 | 16.81±0.67 | 24.64±31.04 |
| **AFP** |  | 11.61±31.75 | 6.47±15.48 | 24.69±52.32*** | 10.63±30.44 | 16.53±37.45 |  | 32.34±90.83 | 32.91±94.05 | 31.83±88.14 | 33.41±93.18 | 31.3±88.81 |
| **FT3** |  | 4.86±0.71 | 4.91±0.71 | 4.64±0.67** | 4.89±0.71 | 4.57±0.67* |  | 6.74±15.62 | 9.37±22.44 | 4.3±1.07* | 8.36±19.84 | 4.13±1.16** |
| **FT4** |  | 15.44±2.36 | 15.63±2.4 | 14.55±1.99*** | 15.53±2.36 | 14.16±1.99** |  | 15.01±3.45 | 15.53±3.28 | 14.51±3.61 | 15.25±2.99 | 14.61±4.18 |
| **GLB** | 29.33±2.78 | 33.93±5.05*** | 33.51±4.48 | 35.01±6.17* | 33.65±4.45 | 35.33±7.22* | 28.73±2.78 | 31.85±6.13*** | 31.01±5.71 | 32.57±6.4* | 31.01±5.71 | 32.57±6.4* |
| **TSH** |  | 2.34±5.02 | 2.39±5.51 | 2.12±1.46 | 2.34±5.18 | 2.35±1.64 |  | 2.63±3.47 | 2.32±3.11 | 2.95±3.83 | 2.55±3 | 2.78±4.24 |
| **Grading of necro-inflammation (%)** | | | | | | | | | | | | |
| **G0** |  | 5.56 |  |  |  |  |  |  |  |  |  |  |
| **G1** |  | 21.01 |  |  |  |  |  |  |  |  |  |  |
| **G2** |  | 33.92 |  |  |  |  |  |  |  |  |  |  |
| **G3** |  | 20.7 |  |  |  |  |  |  |  |  |  |  |
| **G4** |  | 18.81 |  |  |  |  |  |  |  |  |  |  |
| **Staging of liver fibrosis (%)** | | | | | | | | | | | | |
| **S0** |  | 9.39 |  |  |  |  |  |  |  |  |  |  |
| **S1** |  | 34.49 |  |  |  |  |  |  |  |  |  |  |
| **S2** |  | 27.35 |  |  |  |  |  |  |  |  |  |  |
| **S3** |  | 11.43 |  |  |  |  |  |  |  |  |  |  |
| **S4** |  | 17.35 |  |  |  |  |  |  |  |  |  |  |

Note: Values are expressed as mean ± SD.

**Table S2.** Serum bile acid, free fatty acid, and amino acid concentrations in patients with chronic liver disease (CLD) and in normal controls in Cohorts 1 and 2.

| **Dataset** | **Cohort 1 training set** | | | | | | **Cohort 2 validation set** | | | | | |
| --- | --- | --- | --- | --- | --- | --- | --- | --- | --- | --- | --- | --- |
| **Group** | **Control** | **CLD** | **S0-2** | **S3-4** | **Fibrosis** | **Cirrhosis** | **Control** | **CLD** | **S0-2** | **S3-4** | **Fibrosis** | **Cirrhosis** |
| **Number** | 502 | 504 | 349 | 155 | 400 | 104 | 90 | 300 | 134 | 166 | 141 | 159 |
| **GCDCA** | 2.205(1.921, 2.464) | 3.006(2.602, 3.356) | 2.917(2.516, 3.221) | 3.269(2.91, 3.643) | 2.936(2.551, 3.248) | 3.459(3.033, 3.743) |  |  |  |  |  |  |
| **GCA** | 1.544(1.178, 1.864) | 2.597(2.179, 3.09) | 2.461(2.097, 2.907) | 3.046(2.596, 3.562) | 2.505(2.118, 2.947) | 3.352(2.751, 3.652) |  |  |  |  |  |  |
| **CDCA** | 1.836(1.512, 2.173) | 2.055(1.616, 2.389) | 2.004(1.548, 2.353) | 2.193(1.828, 2.527) | 1.994(1.532, 2.353) | 2.3(1.916, 2.695) |  |  |  |  |  |  |
| **GDCA** | 1.401(1.037, 1.724) | 1.928(1.493, 2.39) | 1.993(1.557, 2.399) | 1.825(1.309, 2.321) | 1.993(1.557, 2.399) | 1.783(0.767, 2.169) |  |  |  |  |  |  |
| **DCA** | 1.644(1.292, 1.918) | 1.637(1.198, 1.952) | 1.685(1.375, 1.971) | 1.308(0.637, 1.842) | 1.665(1.31, 1.964) | 1.224(0.59, 1.848) |  |  |  |  |  |  |
| **CA** | 1.22(0.863, 1.593) | 1.678(1.206, 2.143) | 1.546(1.133, 2.031) | 1.875(1.423, 2.334) | 1.562(1.159, 2.05) | 2.056(1.501, 2.521) |  |  |  |  |  |  |
| **UDCA** | 1.272(0.948, 1.615) | 1.351(1.007, 1.724) | 1.302(0.995, 1.679) | 1.465(1.036, 1.887) | 1.308(0.988, 1.679) | 1.558(1.192, 2.089) |  |  |  |  |  |  |
| **GUDCA** | 1.004(0.639, 1.38) | 1.619(1.206, 1.989) | 1.475(1.105, 1.847) | 1.966(1.563, 2.251) | 1.527(1.149, 1.89) | 2.101(1.725, 2.451) |  |  |  |  |  |  |
| **TCDCA** | 0.96(0.706, 1.259) | 2.136(1.68, 2.684) | 2.016(1.607, 2.447) | 2.679(2.125, 3.159) | 2.056(1.628, 2.502) | 2.955(2.338, 3.319) |  |  |  |  |  |  |
| **12-KCDCA** | -0.472(-1.134, 0.251) | -0.405(-1.177, 0.186) | -0.52(-1.231, 0.119) | -0.209(-0.899, 0.344) | -0.387(-1.18, 0.303) | -0.49(-1.135, -0.148) |  |  |  |  |  |  |
| **ACA** | -0.659(-1.35, 0.074) | -0.633(-1.317, 0.069) | -0.661(-1.296, 0.092) | -0.518(-1.321, 0.002) | -0.612(-1.288, 0.155) | -0.712(-1.419, -0.293) |  |  |  |  |  |  |
| **TDCA** | 0.354(-0.111, 0.79) | 1.318(0.754, 1.778) | 1.344(0.821, 1.772) | 1.13(-0.102, 1.798) | 1.349(0.823, 1.78) | 0.998(-0.505, 1.735) |  |  |  |  |  |  |
| **TCA** | 0.162(-0.246, 0.624) | 1.701(1.166, 2.402) | 1.536(1.054, 2.085) | 2.411(1.701, 2.989) | 1.567(1.098, 2.174) | 2.738(2.007, 3.093) | 0.281(-0.216,0.567) | 2.033(1.143,2.897) | 1.648(0.986,2.322) | 2.549(1.531,3.195) | 1.638(0.985,2.284) | 2.703(1.7,3.231) |
| **GHCA** | 0.29(-0.023, 0.575) | 0.996(0.603, 1.424) | 0.93(0.529, 1.333) | 1.252(0.71, 1.547) | 0.973(0.574, 1.376) | 1.269(0.718, 1.535) |  |  |  |  |  |  |
| **HCA** | 0.219(-0.401, 0.627) | 0.74(0.092, 1.177) | 0.705(0.005, 1.152) | 0.863(0.203, 1.293) | 0.725(0.117, 1.172) | 0.924(-0.249, 1.256) |  |  |  |  |  |  |
| **7-KLCA** | -0.03(-0.407, 0.444) | 0.231(-0.25, 0.692) | 0.043(-0.327, 0.647) | 0.357(-0.041, 0.765) | 0.128(-0.304, 0.642) | 0.422(-0.117, 0.846) |  |  |  |  |  |  |
| **7-KDCA** | -0.937(-1.573, -0.355) | -0.687(-1.434, 0.252) | -0.725(-1.462, -0.029) | -0.498(-1.375, 0.455) | -0.715(-1.445, 0.175) | -0.5(-1.25, 0.695) |  |  |  |  |  |  |
| **LCA** | -0.643(-1.134, -0.259) | -0.586(-1.066, -0.064) | -0.602(-1.089, -0.091) | -0.512(-0.966, -0.007) | -0.604(-1.089, -0.109) | -0.347(-0.948, 0.209) |  |  |  |  |  |  |
| **TUDCA** | -0.179(-0.677, 0.068) | 0.489(0.024, 1.005) | 0.376(-0.031, 0.856) | 0.85(0.297, 1.552) | 0.395(-0.031, 0.882) | 1.179(0.453, 1.677) |  |  |  |  |  |  |
| **3-KCA** | -0.912(-1.482, -0.503) | -0.818(-1.434, -0.076) | -0.882(-1.489, -0.225) | -0.68(-1.359, 0.111) | -0.874(-1.49, -0.2) | -0.596(-1.234, 0.144) |  |  |  |  |  |  |
| **23-NDCA** | -0.205(-0.418, -0.023) | -0.239(-0.442, -0.044) | -0.191(-0.397, 0.013) | -0.399(-0.527, -0.16) | -0.191(-0.415, 0.002) | -0.41(-0.552, -0.282) |  |  |  |  |  |  |
| **THCA** | -0.577(-1.07, -0.095) | 0.339(-0.014, 0.881) | 0.247(-0.051, 0.695) | 0.736(0.163, 1.239) | 0.285(-0.031, 0.752) | 0.837(0.251, 1.39) |  |  |  |  |  |  |
| **TLCA** | -0.324(-0.602, -0.089) | 0.005(-0.438, 0.224) | -0.011(-0.446, 0.187) | 0.099(-0.388, 0.411) | -0.021(-0.446, 0.187) | 0.189(-0.321, 0.525) |  |  |  |  |  |  |
| **GLCA** | -0.357(-0.802, 0.067) | 0.062(-0.569, 0.5) | 0.055(-0.449, 0.464) | 0.064(-0.65, 0.631) | 0.07(-0.457, 0.476) | -0.163(-0.675, 0.648) |  |  |  |  |  |  |
| **C8.0** | 0.73(0.453, 1.039) | 0.74(0.469, 1.013) | 0.724(0.452, 0.988) | 0.757(0.497, 1.028) | 0.744(0.468, 1.023) | 0.738(0.484, 0.973) |  |  |  |  |  |  |
| **C10.0** | -0.133(-0.314, 0.028) | -0.2(-0.388, 0.007) | -0.239(-0.411, -0.039) | -0.085(-0.318, 0.092) | -0.228(-0.399, -0.035) | -0.055(-0.295, 0.156) |  |  |  |  |  |  |
| **C12.0** | -0.053(-0.207, 0.116) | -0.118(-0.279, 0.068) | -0.152(-0.292, 0.032) | -0.036(-0.229, 0.133) | -0.147(-0.295, 0.014) | 0.044(-0.181, 0.197) |  |  |  |  |  |  |
| **C14.0** | 0.861(0.7, 0.988) | 0.82(0.671, 0.975) | 0.786(0.639, 0.939) | 0.93(0.758, 1.05) | 0.797(0.638, 0.947) | 0.964(0.798, 1.082) |  |  |  |  |  |  |
| **C14.0.iso** | -0.539(-0.723, -0.402) | -0.757(-1.059, -0.572) | -0.76(-1.058, -0.577) | -0.743(-1.06, -0.558) | -0.77(-1.08, -0.577) | -0.67(-0.947, -0.548) |  |  |  |  |  |  |
| **C14.1.n5c** | -0.475(-0.635, -0.316) | -0.481(-0.735, -0.228) | -0.539(-0.761, -0.336) | -0.288(-0.576, 0.031) | -0.534(-0.762, -0.329) | -0.101(-0.368, 0.252) |  |  |  |  |  |  |
| **C14.1.n5t** | -0.678(-0.966, -0.415) | -0.83(-1.123, -0.5) | -0.878(-1.183, -0.582) | -0.673(-0.988, -0.383) | -0.863(-1.151, -0.58) | -0.59(-0.962, -0.324) |  |  |  |  |  |  |
| **C15.0 iso** | -0.059(-0.367, 0.247) | -0.346(-0.832, -0.027) | -0.36(-0.886, -0.056) | -0.304(-0.645, 0.011) | -0.372(-0.925, -0.056) | -0.233(-0.558, 0.065) |  |  |  |  |  |  |
| **C15.0** | 0.027(-0.122, 0.145) | -0.003(-0.196, 0.114) | -0.012(-0.198, 0.1) | 0.028(-0.187, 0.135) | -0.016(-0.23, 0.102) | 0.063(-0.111, 0.152) |  |  |  |  |  |  |
| **C16.0.iso** | -0.234(-0.597, 0.03) | -0.474(-0.839, -0.158) | -0.493(-0.867, -0.15) | -0.467(-0.771, -0.188) | -0.502(-0.885, -0.16) | -0.435(-0.718, -0.14) |  |  |  |  |  |  |
| **C16.0** | 0.886(0.682, 1.059) | 0.886(0.671, 1.117) | 0.874(0.643, 1.086) | 0.941(0.738, 1.148) | 0.877(0.657, 1.087) | 0.962(0.738, 1.178) |  |  |  |  |  |  |
| **C16.1.n7c** | -0.028(-0.195, 0.112) | 0.111(-0.128, 0.308) | 0.076(-0.154, 0.245) | 0.227(-0.016, 0.408) | 0.088(-0.147, 0.249) | 0.351(0.067, 0.457) |  |  |  |  |  |  |
| **C16.1.n7t** | -0.419(-0.576, -0.312) | -0.551(-0.832, -0.385) | -0.559(-0.856, -0.389) | -0.528(-0.804, -0.376) | -0.556(-0.846, -0.39) | -0.512(-0.798, -0.373) |  |  |  |  |  |  |
| **C16.2.n4c** | -0.69(-0.877, -0.47) | -0.814(-1.048, -0.596) | -0.85(-1.081, -0.622) | -0.718(-0.921, -0.512) | -0.842(-1.071, -0.619) | -0.667(-0.892, -0.511) |  |  |  |  |  |  |
| **C17.0.iso** | -0.133(-0.494, 0.137) | -0.355(-0.755, -0.005) | -0.364(-0.768, -0.01) | -0.275(-0.749, 0.004) | -0.364(-0.798, -0.008) | -0.224(-0.548, 0.023) |  |  |  |  |  |  |
| **C17.0** | 0.333(0.101, 0.514) | 0.232(-0.029, 0.464) | 0.219(-0.04, 0.442) | 0.272(0.004, 0.48) | 0.228(-0.04, 0.443) | 0.249(0.016, 0.504) |  |  |  |  |  |  |
| **C17.1.n7c** | 0(-0.15, 0.117) | -0.008(-0.22, 0.17) | -0.053(-0.227, 0.122) | 0.134(-0.161, 0.284) | -0.04(-0.232, 0.126) | 0.196(-0.014, 0.312) |  |  |  |  |  |  |
| **C18.0.iso** | -0.387(-0.615, -0.223) | -0.513(-0.744, -0.314) | -0.528(-0.729, -0.329) | -0.471(-0.778, -0.286) | -0.528(-0.764, -0.328) | -0.441(-0.623, -0.264) |  |  |  |  |  |  |
| **C18.0** | 0.84(0.684, 0.968) | 0.81(0.65, 0.948) | 0.796(0.627, 0.933) | 0.835(0.682, 0.98) | 0.81(0.638, 0.946) | 0.812(0.682, 0.98) |  |  |  |  |  |  |
| **C18.1.n9c** | 1.049(0.954, 1.141) | 1.088(0.967, 1.195) | 1.072(0.964, 1.174) | 1.151(0.987, 1.237) | 1.072(0.963, 1.182) | 1.178(1.039, 1.265) |  |  |  |  |  |  |
| **C18.1.n9t** | 0.467(0.375, 0.541) | 0.133(-0.105, 0.283) | 0.112(-0.145, 0.257) | 0.179(0.015, 0.346) | 0.107(-0.145, 0.254) | 0.249(0.074, 0.365) |  |  |  |  |  |  |
| **C18.2.n6c** | 1.153(0.984, 1.288) | 1.124(0.942, 1.295) | 1.096(0.935, 1.248) | 1.227(0.999, 1.369) | 1.099(0.935, 1.267) | 1.264(1.061, 1.428) |  |  |  |  |  |  |
| **C18.2.n6t** | 1.892(1.353, 2.215) | -1.943(-2.788, -1.209) | -2.087(-2.971, -1.354) | -1.45(-2.188, -0.795) | -2.027(-2.876, -1.328) | -1.03(-2.1, -0.22) | 0.397(-0.411,1.16) | -1.831(-2.756,-0.646) | -2.151(-3.083,-1.119) | -1.464(-2.286,-0.336) | -2.083(-3.023,-1.118) | -1.457(-2.301,-0.32) |
| **C18.3.n3c** | -0.067(-0.225, 0.116) | -0.125(-0.314, 0.076) | -0.153(-0.348, 0.024) | 0.02(-0.246, 0.181) | -0.15(-0.348, 0.036) | 0.039(-0.194, 0.195) |  |  |  |  |  |  |
| **C18.3.n6c** | 0.463(0.274, 0.627) | 0.275(0.045, 0.486) | 0.225(0.002, 0.431) | 0.42(0.204, 0.623) | 0.237(0.02, 0.442) | 0.526(0.288, 0.726) |  |  |  |  |  |  |
| **C19.0** | -0.584(-0.96, -0.369) | -0.692(-1.054, -0.415) | -0.702(-1.054, -0.417) | -0.641(-1.052, -0.409) | -0.711(-1.07, -0.421) | -0.613(-0.962, -0.364) |  |  |  |  |  |  |
| **C19.1.n9c** | -0.004(-0.247, 0.177) | -0.001(-0.294, 0.282) | -0.057(-0.322, 0.229) | 0.139(-0.189, 0.382) | -0.036(-0.32, 0.23) | 0.248(-0.069, 0.43) |  |  |  |  |  |  |
| **C20.0** | 0(-0.438, 0.19) | 0.014(-0.311, 0.206) | 0.013(-0.307, 0.209) | 0.015(-0.347, 0.194) | 0.014(-0.315, 0.209) | 0.011(-0.194, 0.194) |  |  |  |  |  |  |
| **C20.1.n9c** | 0.054(-0.108, 0.21) | 0.127(-0.044, 0.325) | 0.091(-0.068, 0.264) | 0.213(0.024, 0.437) | 0.102(-0.064, 0.275) | 0.314(0.123, 0.473) |  |  |  |  |  |  |
| **C20.2.n6c** | 0.299(0.146, 0.45) | 0.297(0.128, 0.476) | 0.251(0.104, 0.442) | 0.382(0.198, 0.55) | 0.259(0.111, 0.443) | 0.459(0.242, 0.617) |  |  |  |  |  |  |
| **C20.3.n6c** | 0.071(-0.03, 0.208) | 0.013(-0.104, 0.153) | 0(-0.124, 0.13) | 0.065(-0.069, 0.186) | 0.004(-0.113, 0.135) | 0.067(-0.058, 0.202) |  |  |  |  |  |  |
| **C20.4.n6c** | 0.351(0.276, 0.447) | 0.262(0.155, 0.395) | 0.254(0.155, 0.385) | 0.278(0.165, 0.416) | 0.261(0.159, 0.391) | 0.272(0.149, 0.416) |  |  |  |  |  |  |
| **C20.5.n3c** | 0.252(0.096, 0.411) | 0.241(0.049, 0.428) | 0.245(0.029, 0.436) | 0.219(0.077, 0.396) | 0.244(0.047, 0.434) | 0.214(0.077, 0.393) |  |  |  |  |  |  |
| **C22.1.n9c** | -0.54(-0.741, -0.326) | -0.591(-0.81, -0.35) | -0.628(-0.834, -0.414) | -0.45(-0.703, -0.271) | -0.616(-0.824, -0.4) | -0.387(-0.634, -0.249) |  |  |  |  |  |  |
| **C22.2.n6c** | 0.997(0.868, 1.133) | 1.042(0.879, 1.2) | 1.017(0.853, 1.139) | 1.166(0.965, 1.349) | 1.022(0.856, 1.152) | 1.256(1.004, 1.433) |  |  |  |  |  |  |
| **C22.4.n6c** | 1.238(1.115, 1.398) | 1.229(1.061, 1.414) | 1.198(1.038, 1.364) | 1.332(1.173, 1.478) | 1.212(1.043, 1.368) | 1.395(1.187, 1.52) |  |  |  |  |  |  |
| **C22.5.n6c** | -0.086(-0.208, 0.048) | -0.127(-0.274, 0.025) | -0.15(-0.283, 0.005) | -0.086(-0.221, 0.073) | -0.135(-0.276, 0.01) | -0.058(-0.239, 0.119) |  |  |  |  |  |  |
| **C22.5.n3c** | 0.595(0.45, 0.744) | 0.562(0.39, 0.722) | 0.54(0.376, 0.714) | 0.601(0.435, 0.741) | 0.547(0.382, 0.715) | 0.62(0.452, 0.757) |  |  |  |  |  |  |
| **C24.0** | -1.458(-2.287, -1.13) | -1.499(-2.324, -1.096) | -1.467(-2.29, -1.052) | -1.607(-2.388, -1.224) | -1.478(-2.292, -1.062) | -1.731(-2.457, -1.244) |  |  |  |  |  |  |
| **C24.1.n9c** | -0.643(-0.821, -0.48) | -0.577(-0.748, -0.423) | -0.607(-0.778, -0.458) | -0.509(-0.676, -0.386) | -0.605(-0.777, -0.442) | -0.474(-0.643, -0.364) |  |  |  |  |  |  |
| **C22.6.n3c** | 0.402(0.252, 0.563) | 0.396(0.223, 0.557) | 0.391(0.225, 0.547) | 0.417(0.202, 0.573) | 0.392(0.222, 0.547) | 0.438(0.228, 0.578) |  |  |  |  |  |  |
| **C16.1.n9c** | 0.577(0.163, 0.976) | 0.246(-0.298, 0.683) | 0.216(-0.263, 0.622) | 0.362(-0.496, 0.862) | 0.246(-0.218, 0.683) | 0.238(-0.649, 0.698) |  |  |  |  |  |  |
| **Ala** | 2.443(2.382, 2.654) | 2.569(2.479, 2.65) | 2.566(2.485, 2.646) | 2.57(2.472, 2.655) | 2.568(2.483, 2.649) | 2.576(2.462, 2.65) |  |  |  |  |  |  |
| **Arg** | 2.03(1.877, 2.141) | 1.982(1.92, 2.047) | 1.981(1.921, 2.039) | 1.988(1.919, 2.064) | 1.982(1.924, 2.039) | 1.985(1.902, 2.072) |  |  |  |  |  |  |
| **Asn** | 1.485(1.359, 1.671) | 1.725(1.658, 1.801) | 1.719(1.653, 1.778) | 1.764(1.677, 1.841) | 1.719(1.653, 1.784) | 1.772(1.692, 1.845) |  |  |  |  |  |  |
| **Cit** | 1.461(1.354, 1.614) | 1.493(1.385, 1.596) | 1.471(1.361, 1.56) | 1.572(1.46, 1.697) | 1.476(1.375, 1.572) | 1.615(1.489, 1.712) |  |  |  |  |  |  |
| **Gln** | 2.933(2.857, 3.018) | 2.935(2.874, 2.989) | 2.934(2.877, 2.979) | 2.942(2.863, 3.014) | 2.934(2.875, 2.981) | 2.951(2.863, 3.026) |  |  |  |  |  |  |
| **Glu** | 1.7(1.628, 1.934) | 1.804(1.729, 1.919) | 1.805(1.729, 1.918) | 1.804(1.729, 1.925) | 1.816(1.734, 1.922) | 1.776(1.713, 1.893) |  |  |  |  |  |  |
| **Gly** | 2.244(2.098, 2.345) | 2.454(2.385, 2.524) | 2.451(2.384, 2.522) | 2.463(2.389, 2.529) | 2.446(2.384, 2.518) | 2.496(2.411, 2.543) |  |  |  |  |  |  |
| **His** | 1.879(1.803, 1.952) | 2.01(1.951, 2.06) | 2.008(1.954, 2.059) | 2.014(1.945, 2.061) | 2.006(1.954, 2.058) | 2.018(1.942, 2.077) |  |  |  |  |  |  |
| **Ile** | 1.978(1.868, 2.054) | 1.884(1.805, 1.974) | 1.888(1.807, 1.977) | 1.867(1.785, 1.965) | 1.889(1.81, 1.979) | 1.839(1.75, 1.927) |  |  |  |  |  |  |
| **Leu** | 1.962(1.729, 2.214) | 2.041(1.952, 2.152) | 2.051(1.964, 2.159) | 2.007(1.917, 2.137) | 2.054(1.963, 2.159) | 1.994(1.874, 2.104) |  |  |  |  |  |  |
| **Lys** | 2.207(2.036, 2.394) | 2.131(2.042, 2.218) | 2.143(2.055, 2.221) | 2.105(1.985, 2.208) | 2.143(2.053, 2.223) | 2.085(1.96, 2.185) |  |  |  |  |  |  |
| **Met** | 1.135(0.994, 1.233) | 1.422(1.362, 1.509) | 1.404(1.347, 1.479) | 1.527(1.416, 1.644) | 1.409(1.352, 1.485) | 1.549(1.462, 1.73) |  |  |  |  |  |  |
| **Orn** | 1.822(1.626, 1.903) | 1.82(1.687, 1.927) | 1.794(1.668, 1.908) | 1.869(1.739, 1.977) | 1.8(1.675, 1.909) | 1.887(1.726, 2.058) |  |  |  |  |  |  |
| **Phe** | 1.572(1.511, 1.629) | 1.858(1.788, 1.944) | 1.844(1.783, 1.907) | 1.924(1.836, 2) | 1.844(1.784, 1.92) | 1.958(1.876, 2.042) |  |  |  |  |  |  |
| **Pro** | 2.284(2.046, 2.383) | 2.246(2.164, 2.326) | 2.231(2.151, 2.319) | 2.281(2.216, 2.355) | 2.233(2.159, 2.32) | 2.301(2.234, 2.355) |  |  |  |  |  |  |
| **Ser** | 2(1.917, 2.1) | 2.198(2.137, 2.269) | 2.193(2.135, 2.256) | 2.232(2.158, 2.292) | 2.194(2.136, 2.259) | 2.236(2.16, 2.304) |  |  |  |  |  |  |
| **Thr** | 2.018(1.91, 2.119) | 2.135(2.071, 2.201) | 2.134(2.072, 2.198) | 2.14(2.065, 2.225) | 2.134(2.073, 2.2) | 2.14(2.058, 2.211) |  |  |  |  |  |  |
| **Trp** | 1.518(1.436, 1.575) | 1.746(1.681, 1.812) | 1.748(1.691, 1.805) | 1.731(1.64, 1.83) | 1.751(1.693, 1.812) | 1.7(1.605, 1.816) |  |  |  |  |  |  |
| **Tyr** | 1.864(1.819, 2.053) | 1.935(1.86, 2.019) | 1.908(1.846, 1.972) | 2.044(1.946, 2.146) | 1.919(1.852, 1.99) | 2.084(1.963, 2.176) | 1.86(1.815,1.895) | 1.976(1.843,2.129) | 1.904(1.778,2.008) | 2.047(1.937,2.16) | 1.906(1.779,2.021) | 2.056(1.938,2.165) |
| **Val** | 2.447(2.38, 2.516) | 2.39(2.306, 2.465) | 2.405(2.323, 2.472) | 2.359(2.243, 2.443) | 2.408(2.325, 2.475) | 2.295(2.201, 2.41) | 2.476(2.37,2.555) | 2.355(2.244,2.505) | 2.353(2.269,2.507) | 2.355(2.231,2.502) | 2.363(2.271,2.507) | 2.347(2.228,2.491) |
| **alpha.AAA** | 0.492(0.035, 0.792) | 0.577(0.564, 1.001) | 0.577(0.565, 1.001) | 0.576(0.564, 1.002) | 0.576(0.565, 1.001) | 0.577(0.563, 1.002) |  |  |  |  |  |  |
| **Creatinine** | 1.786(1.644, 1.916) | 1.9(1.752, 2.162) | 1.904(1.757, 2.168) | 1.894(1.734, 2.126) | 1.892(1.754, 2.162) | 1.955(1.734, 2.127) |  |  |  |  |  |  |
| **Histamine** | -0.611(-0.775, -0.251) | -0.357(-0.361, -0.118) | -0.357(-0.361, -0.117) | -0.357(-0.36, -0.118) | -0.357(-0.361, -0.118) | -0.357(-0.36, -0.118) |  |  |  |  |  |  |
| **Kynurenine** | -0.076(-0.322, 0.076) | 0.349(0.235, 0.446) | 0.342(0.232, 0.438) | 0.374(0.249, 0.472) | 0.35(0.235, 0.442) | 0.342(0.241, 0.471) |  |  |  |  |  |  |
| **Met.SO** | -0.01(-0.102, 0.139) | -0.025(-0.205, 0.086) | -0.026(-0.226, 0.061) | -0.002(-0.119, 0.132) | -0.026(-0.212, 0.063) | 0.024(-0.127, 0.178) |  |  |  |  |  |  |
| **PEA** | -1.745(-1.854, -1.602) | -1.649(-1.867, -1.591) | -1.652(-1.857, -1.592) | -1.642(-1.883, -1.583) | -1.65(-1.855, -1.59) | -1.647(-2.014, -1.598) |  |  |  |  |  |  |
| **Putrescine** | -0.747(-1.319, -0.396) | -0.906(-1.045, -0.768) | -0.912(-1.061, -0.772) | -0.896(-1.005, -0.73) | -0.91(-1.053, -0.772) | -0.899(-1.004, -0.724) |  |  |  |  |  |  |
| **Sarcosine** | 1.176(0.84, 1.24) | 1.259(1.172, 1.344) | 1.263(1.178, 1.343) | 1.24(1.165, 1.347) | 1.26(1.175, 1.345) | 1.251(1.157, 1.328) |  |  |  |  |  |  |
| **Serotonin** | -0.796(-1.398, -0.489) | -0.457(-0.706, -0.263) | -0.414(-0.661, -0.259) | -0.608(-0.911, -0.331) | -0.417(-0.66, -0.255) | -0.663(-1.015, -0.439) |  |  |  |  |  |  |
| **Spermidine** | -0.979(-1.143, -0.903) | -0.648(-0.743, -0.559) | -0.656(-0.739, -0.562) | -0.628(-0.756, -0.521) | -0.656(-0.744, -0.565) | -0.59(-0.739, -0.484) |  |  |  |  |  |  |
| **Spermine** | -0.478(-0.52, -0.462) | -0.644(-0.667, -0.551) | -0.643(-0.667, -0.551) | -0.646(-0.667, -0.552) | -0.645(-0.667, -0.553) | -0.643(-0.666, -0.548) |  |  |  |  |  |  |
| **Taurine** | 1.784(1.676, 1.904) | 1.913(1.801, 2.072) | 1.937(1.819, 2.094) | 1.87(1.743, 1.995) | 1.933(1.816, 2.088) | 1.83(1.696, 1.981) |  |  |  |  |  |  |

Note: Data are represented as median (25% quantile, 75% quantile). All concentration values were log10 transformed. The concentration unit for BAs is ng/ml, for FFAs is µg/ml, and for AAs is µM.

**Abbreviations:** C8:0, Caprylic acid; C10:0, Capric acid; C12:0, Lauric acid; C14:0, Myristic acid; C14:0iso, 12-Methyltridecanoic acid; C14:1n5c, Myristoleic acid; C14:1n5t, Myristelaidic acid; C15:0, Pentadecanoic acid; C15:0 iso, 13-Methylmyristic acid; C16:0, Palmitic acid; C16:0iso, 14-methylpentadecanoic acid; C16:1n7c, Palmitoleic acid; C16:1n7t, Palmitelaidic acid; C16:1n9c, cis-7 hexadecenoic acid; C16:2n4c, Hexadecadienoic acid; C17:0, Margaric acid; C17:0iso, 15-Methylpalmitic acid; C17:1n7c, Heptadecenoic acid; C18:0, Stearic acid; C18:0iso, 16-Methylmargaric acid; C18:1n9c, Oleic acid; C18:1n9t, Elaidic acid; C18:2n6c, Linoleic acid; C18:2n6t, Linolelaidic acid; C18:3n3c, α-Linolenic acid; C18:3n6c, γ-Linolenic acid; C19:0, Nonadecanoic acid; C19:1n9c, Nonadecenoic acid; C20:0, Arachidic acid; C20:1n9c, Eicosenoic acid; C20:2:n6c, Eicosadienoic acid; C20:3n6c, dihomo-γ-linolenic acid; C20:4n6c, Arachidonic acid; C20:5n3c, Eicosapentaenoic acid; C22:1n9c, Erucic acid; C22:2n6c, Docosadienoic acid; C22:4n6c, Docosatetraenoic acid; C22:5n3c, Docosapentaenoic acid; C22:5n6c, Docosapentaenoic acid; C22:6n3c, Docosahexaenoic acid; C24:0, Lignoceric acid; C24:1n9c, Nervonic acid; GCDCA, Glycochenodeoxycholate; GCA, Glycocholate; CDCA, Chenodeoxycholate; GDCA, Glycodeoxycholate; DCA, Deoxycholate; CA, Cholate; UDCA, Ursodeoxycholate; GUDCA, Glycoursodeoxycholate; TCDCA, Taurochenodeoxycholate; 12-KCDCA, 12-ketochenodeoxycholate; ACA, Apocholate; TDCA, Tautodeoxycholate; TCA, Taurocholate; GHCA, Glyco-hyocholate; HCA, Hyocholate; 7-KLCA, 7-Ketolithocholate; 7-KDCA, 7-Ketodeoxycholate; LCA, Lithocholate; TUDCA, Tauroursodeoxycholate; 3-KCA, 3-Ketocholate; 23-NDCA, 23-Nordeoxycholate; THCA, Taurohyocholate; TLCA, Taurolitholate; GLCA, Glycolithocholate; Ala, Alanine; Arg, Arginine; Asn, Asparagine; Cit, Citrulline; Gln, Glutamine; Glu, Glutamate; Gly, Glycine; His, Histidine; Ile, Isoleucine; Leu, Leucine; Lys, Lysine; Met, Methionine; Orn, Ornithine; Phe, Phenylalanine; Pro, Proline; Ser, Serine; Thr, Threonine; Trp, Tryptophan; Tyr, Tyrosine; Val, Valine; alpha.AAA, alpha-Aminoadipic acid; Met.SO, Methioninesulfoxide; PEA, Phenylethylamine

**Table S3**. Results for measurement of the metabolite marker panel, APRI, FIB-4, and ALT/AST ratio in the prediction of liver fibrosis using the optimal cut-off values generated using the cohort specific data from this study.

|  | **Cohort 1 training set** | | | **Cohort 2 validation set** | | |
| --- | --- | --- | --- | --- | --- | --- |
|  | **CLD vs. controls** | **Fibrosis vs. Cirrhosis** | **S0-2 vs. S34** | **CLD vs. controls** | **Fibrosis vs. Cirrhosis** | **S0-2 vs. S34** |
| **Metabolite marker panel** | | | | | | |
| AUROC (95% CI) | 0.997 (0.993-1) | 0.941 (0.914-0.964) | 0.918 (0.889-0.946) | 0.977 (0.963-0.988) | 0.844 (0.797-0.884) | 0.807 (0.756-0.852) |
| AUPR (95% CI) | 0.994 (0.986-1) | 0.87 (0.824-0.913) | 0.892 (0.854-0.925) | 0.993 (0.989-0.997) | 0.827 (0.761-0.884) | 0.817 (0.764-0.866) |
| Cutoff values**^†^** | 0.43 | 0.010 | -0.115 | 0.434 | 0.010 | -0.115 |
| Sensitivity (%) | 98.4 | 87.0 | 86.7 | 92.2 | 68.8 | 68.9 |
| Specificity (%) | 99.0 | 90.4 | 90.5 | 94.4 | 81.6 | 76.1 |
| **FIB-4** | | | | | | |
| AUROC (95% CI) | 0.848 (0.823-0.87) | 0.869 (0.829-0.906) | 0.802 (0.762-0.844) | 0.707 (0.652-0.762) | 0.758 (0.692-0.815) | 0.739 (0.68-0.798) |
| AUPR (95% CI) | 0.863 (0.84-0.883) | 0.725 (0.657-0.79) | 0.707 (0.651-0.761) | 0.897 (0.873-0.918) | 0.726 (0.657-0.794) | 0.726 (0.66-0.795) |
| Cutoff values**^†^** | 0.843 | 2.387 | 1.841 | 0.843 | 2.387 | 1.841 |
| Sensitivity (%) | 70.6 | 74.8 | 61.9 | 90.7 | 75.0 | 77.0 |
| Specificity (%) | 84.4 | 86.7 | 84.8 | 9.0 | 69.5 | 53.0 |
| **APRI** | | | | | | |
| AUROC (95% CI) | 0.973 (0.965-0.981) | 0.698 (0.644-0.752) | 0.647 (0.595-0.698) | 0.879 (0.841-0.915) | 0.608 (0.534-0.671) | 0.595 (0.529-0.669) |
| AUPR (95% CI) | 0.977 (0.969-0.983) | 0.416 (0.345-0.497) | 0.492 (0.434-0.554) | 0.958 (0.942-0.972) | 0.53 (0.474-0.605) | 0.542 (0.488-0.614) |
| Cutoff values**^†^** | 0.154 | 0.825 | 0.336 | 0.154 | 0.825 | 0.336 |
| Sensitivity (%) | 89.6 | 47.0 | 74.6 | 85.9 | 26.6 | 67.4 |
| Specificity (%) | 95.1 | 84.4 | 49.0 | 75.3 | 81.6 | 52.2 |
| **AST/ALT** | | | | | | |
| AUROC (95% CI) | 0.665 (0.631-0.697) | 0.815 (0.766-0.862) | 0.714 (0.668-0.759) | 0.603 (0.54-0.657) | 0.684 (0.619-0.75) | 0.667 (0.597-0.728) |
| AUPR (95% CI) | 0.714 (0.685-0.747) | 0.579 (0.496-0.674) | 0.582 (0.516-0.654) | 0.849 (0.819-0.875) | 0.641 (0.573-0.721) | 0.648 (0.583-0.727) |
| Cutoff values**^†^** | 0.519 | 0.921 | 0.686 | 0.519 | 0.921 | 0.686 |
| Sensitivity (%) | 41.4 | 63.5 | 60.8 | 13.0 | 71.9 | 83.0 |
| Specificity (%) | 88.3 | 90.9 | 73.1 | 100.0 | 51.1 | 32.1 |
| **Comparison of AUROC** | | | | | | |
| Metabolite marker panel versus FIB-4 | p<0.001 | p<0.001 | p<0.001 | p<0.001 | p<0.001 | 0.01 |
| Metabolite marker panel versus APRI | p<0.001 | p<0.001 | p<0.001 | p<0.001 | p<0.001 | p<0.001 |
| Metabolite marker panel versus AST/ALT | p<0.001 | p<0.001 | p<0.001 | p<0.001 | p<0.001 | p<0.001 |

^†^Cut-off values were determined to maximize the sum of sensitivity and specificity in the training data set.

APRI, AST-to-platelet ratio index; AST/ALT; aspartate transaminase/alanine transaminase ratio; FIB-4, fibrosis-4 index; AUROC, area under the receiver operating characteristic curve; AUPR, area under the precision-recall (PR) curve

**Table S4**. Logistic regression analysis of metabolite marker panel-based RF-score to discriminate patients with fibrosis from patients with cirrhosis and S0-2 with S3-4 adjusting with potential confounding variables.

|  | **Estimate** | **SE** | **P value** |
| --- | --- | --- | --- |
| **S0-2 vs. S3-4** | | | |
| RF-score | 0.805 | 0.126 | **<0.001** |
| log_10_(HBV DNA) | -0.049 | 0.085 | 0.566 |
| necroinflammation | 1.304 | 0.263 | <0.001 |
| HBeAb_bin | -0.171 | 0.347 | 0.623 |
| HBeAg_bin | -0.670 | 0.379 | 0.077 |
| BMI | 0.050 | 0.055 | 0.363 |
| PLT | -0.012 | 0.003 | 0.001 |
| PT | 0.122 | 0.117 | 0.298 |
| ALB | -0.003 | 0.037 | 0.936 |
| DBIL | 0.019 | 0.020 | 0.334 |
| IBIL | -0.020 | 0.020 | 0.305 |
| Medication (Entecavir) | 0.489 | 0.436 | 0.262 |
| **Fibrosis vs. Cirrhosis** | | | |
| RF-score | 0.755 | 0.158 | **<0.001** |
| log_10_(HBV DNA) | -0.075 | 0.098 | 0.441 |
| necroinflammation | 0.253 | 0.286 | 0.377 |
| HBeAb_bin | -0.598 | 0.412 | 0.147 |
| HBeAg_bin | 0.024 | 0.438 | 0.956 |
| BMI | 0.051 | 0.062 | 0.408 |
| PLT | -0.012 | 0.004 | 0.003 |
| PT | 0.275 | 0.131 | 0.036 |
| ALB | -0.028 | 0.040 | 0.485 |
| DBIL | 0.005 | 0.018 | 0.785 |
| IBIL | -0.024 | 0.021 | 0.249 |
| Medication (Entecavir) | 0.719 | 0.433 | 0.097 |

**Table S5.** Net reclassification improvement and integral discriminant improvement analyses comparing RF and other clinical indexes on validation sets.

|  |  | **NRI(Categorical) [95% CI]** | **P-value** | **NRI(Continuous) [95% CI]** | **P-value** | **IDI [95% CI]** | **P-value** |
| --- | --- | --- | --- | --- | --- | --- | --- |
| **CLD v.s. Control** | RF v.s. FIB4 | 1.404 [ 1.2685 - 1.5394 ] | < 0.001 | 1.3817 [ 1.2313 - 1.5322 ] | < 0.001 | 0.6244 [ 0.5647 - 0.6842 ] | < 0.001 |
|  | RF v.s. APRI | 1.0837 [ 0.9388 - 1.2285 ] | < 0.001 | 0.816 [ 0.6346 - 0.9975 ] | < 0.001 | 0.3987 [ 0.3324 - 0.4651 ] | < 0.001 |
|  | RF v.s. AST/ALT ratio | 1.7089 [ 1.5864 - 1.8314 ] | < 0.001 | 1.6938 [ 1.5615 - 1.8261 ] | < 0.001 | 0.7279 [ 0.6741 - 0.7817 ] | < 0.001 |
| **Fibrosis v.s. Cirrhosis** | RF v.s. FIB4 | 0.7891 [ 0.5925 - 0.9857 ] | < 0.001 | 0.8487 [ 0.6323 - 1.0652 ] | < 0.001 | 0.1894 [ 0.1411 - 0.2377 ] | < 0.001 |
|  | RF v.s. APRI | 0.9893 [ 0.7936 - 1.1849 ] | < 0.001 | 1.0829 [ 0.8825 - 1.2833 ] | < 0.001 | 0.3574 [ 0.3004 - 0.4143 ] | < 0.001 |
|  | RF v.s. AST/ALT ratio | 0.9496 [ 0.7527 - 1.1464 ] | < 0.001 | 0.9709 [ 0.7616 - 1.1801 ] | < 0.001 | 0.2729 [ 0.2179 - 0.3278 ] | < 0.001 |
| **S0-2 v.s. S34** | RF v.s. FIB4 | 0.6329 [ 0.4356 - 0.8303 ] | < 0.001 | 0.6331 [ 0.4091 - 0.857 ] | < 0.001 | 0.1281 [ 0.0831 - 0.173 ] | < 0.001 |
|  | RF v.s. APRI | 0.8927 [ 0.6871 - 1.0983 ] | < 0.001 | 0.8855 [ 0.6724 - 1.0985 ] | < 0.001 | 0.2713 [ 0.2185 - 0.3241 ] | < 0.001 |
|  | RF v.s. AST/ALT ratio | 0.8108 [ 0.6079 - 1.0137 ] | < 0.001 | 0.8557 [ 0.6409 - 1.0705 ] | < 0.001 | 0.1993 [ 0.1479 - 0.2508 ] | < 0.001 |

Note:

NRI(Categorical) = event NRI + nonevent NRI = [Pr(up|event) - Pr(down|event)] + [Pr(down|nonevent) - Pr(up|nonevent)] = [(number of events classified up - number of events classified down)/number of events] + [(number of nonevents classified down - number of nonevents classified up)/number of nonevents]

NRI(Continuous) = event NRI + nonevent NRI = [Pr(higher|event) - Pr(lower|event)] + [Pr(lower|nonevent) - Pr(higher|nonevent)] = [(number of events with increased predicted risk - number of events with decreased predicted risk)/number of events] + [(number of nonevents with decreased predicted risk - number of nonevents with increased predicted risk)/number of nonevents]

For categorical NRI and continuous NRI, the theoretical range is –2 to 2.

IDI was used to compare mean differences in predicted risks of subjects with and without the event between RF models and clinical indexes.
